# The Use of a Digital Social Forum in a Parkinson’s Disease Risk Cohort: A Thematic Analysis of Forum Messages

**DOI:** 10.1155/padi/7537110

**Published:** 2026-02-13

**Authors:** Laura J. Smith, Janusiya Jeyaganesh, Xiancheng Li, Aneet Gill, Anette Schrag, Brook Huxford, Cristina Simonet, Laura Pérez-Carbonell, Alastair J. Noyce, Anna De Simoni

**Affiliations:** ^1^ Centre for Preventive Neurology, Wolfson Institute of Population Health, Queen Mary University of London, London, UK, qmul.ac.uk; ^2^ School of Business and Management, Queen Mary University of London, London, UK, qmul.ac.uk; ^3^ Institute of Clinical Trials and Methodology, University College London, London, UK, ucl.ac.uk; ^4^ Queen Square Institute of Neurology, University College London, London, UK, ucl.ac.uk; ^5^ Sleep Disorders Centre, Guy’s and St Thomas’ NHS Foundation Trust, London, UK, nhs.uk; ^6^ Centre for Primary Care, Wolfson Institute of Population Health, Queen Mary University of London, London, UK, qmul.ac.uk

**Keywords:** online forum, Parkinson’s disease, prodromal, risk, social engagement, thematic analysis

## Abstract

**Background:**

Reduced social engagement is associated with increased risk of incident Parkinson’s disease (PD). Online peer support provides opportunities to develop new social connections. A digital social forum was recently embedded within PREDICT‐PD, an online UK cohort study that stratifies participants for risk of future PD, to explore the feasibility of digital social engagement as an intervention to modify PD risk.

**Objective:**

This study reports on the content of messages exchanged on the forum to better understand how this was used and experienced.

**Methods:**

364 public posts from 218 distinct users were analysed using thematic analysis.

**Results:**

Members created a sense of community through disclosing personal information and reaching out to others. Experiences were shared in relation to symptom appraisal, emotional impacts and routes to diagnosis. Practical advice, resources and information were exchanged to aid symptom management and proactive lifestyle changes. Users discussed their aspirations for timely diagnosis and treatment within healthcare, further research funding to aid prevention and treatment, and greater awareness of PD within society. Technical issues with the forum were reported, and accessibility was viewed as a potential barrier.

**Conclusions:**

The online forum provided a peer support environment for people with similar health experiences to connect, exchange information and emotional support, and engage in discussions around political and social issues unique to PD. This highlights the potential of leveraging online peer support to promote social engagement in prodromal PD. Further research is needed to examine the effect on PD risk and develop accessible technologies.

## 1. Introduction

Parkinson’s disease (PD) is the second most common neurodegenerative condition, affecting 1%–2% of the population ≥ 65 years of age [1]. The prodromal phase of the disease is characterised by several non‐motor symptoms, which can precede the onset of classic motor symptoms for decades [2]. These include isolated REM (rapid eye movement) sleep behaviour disorder (iRBD), anosmia and ageusia, cognitive dysfunction, mental health disturbances and dysautonomia [3, 4].

This prodromal phase provides a window of opportunity for early intervention to slow progression and preserve compensatory mechanisms, thereby limiting disability [5]. Successful prevention and disease modification are likely to require a multifaceted approach [6], and lifestyle interventions offer a promising avenue [7]. Recent studies have shown that regular exercise and a Mediterranean diet can slow progression from prodromal to manifest PD risk [7]. Social engagement is also thought to be neuroprotective in healthy older adults [8–10]. Meanwhile, reduced social engagement is associated with an increased risk of incident PD and dementia [11], reduced quality of life and pathological changes in the PD brain [12, 13]. To date, social interventions have not been explored within prodromal PD.

One way in which people can stay socially connected is through peer support. Peer support can be defined as the reciprocal exchange of emotional, appraisal and informational assistance with others who share a similar health condition or life experience [14, 15]. Peers can share experiential knowledge based on their personal experiences to aid understanding and give a sense of empowerment [16]. Moreover, developing social connections can decrease feelings of loneliness and isolation, and improve mood [17].

With the growing availability and access to the internet, online peer support programs have become increasingly common [18]. Compared with local community groups, online peer support can be accessed from home, by a larger and more diverse network, anonymously and at any time. Users can also choose whether to actively participate or adopt a more passive position (e.g., reading online communications) [19]. The beneficial impacts of online peer support have already been reported in PD, with digital platforms widening opportunities for social engagement and serving as a support system for people with PD to connect with one another [20], though PD symptoms hindered technology usage amongst some users [16]. This parallels findings from digital studies with healthy older adults, for whom social use of technology predicted greater well‐being over time by reducing loneliness and increasing the breadth of community engagement [21].

Online peer support communities have not yet been explored amongst individuals at‐risk of developing PD. Currently, there is little support available for people with prodromal PD, but several strands of research indicate these individuals may benefit from increased support. Firstly, those in the prodromal phase can present with a range of physical and mental health complaints severe enough for them to visit their general practitioner more frequently than controls [22, 23]. Secondly, when disclosed, the diagnostic uncertainty and lack of available therapeutic options can be distressing for affected at‐risk individuals [24]. A quarter of patients with PD would thus endorse psychological support after risk disclosure during the prodromal phase [25]. Online peer support communities may therefore provide an important function of support for individuals with increased risk of PD.

PREDICT‐PD [26] is a landmark project using online recruitment and assessments to identify people at‐risk of PD. Currently, more than 17,000 people have registered across the UK. A digital forum was recently embedded within the PREDICT‐PD website to enable social engagement and explore the feasibility of digital social engagement as an intervention to modify PD risk [27]. Here, we aim to explore the content of messages exchanged to better understand how the digital social forum was used and experienced.

## 2. Methods

### 2.1. Data Collection

Data were messages posted to an asynchronous moderated discussion forum embedded within the PREDICT‐PD portal based in the UK. The online forum was made public for a period of four months and accessed by 218 users who posted under anonymised usernames. Users could choose to either write posts publicly or privately (shared between two users). Only posts that were shared publicly were collected and analysed. Patient and public involvement (PPI) informed the design of the forum, including instating a full‐time moderator to manage the forum and weekly engagement activities posted by the research team (including PD‐related research, facts and queries) to stimulate discussions. Example activity topics included the following: Rises in the prevalence of Parkinson’s, Parkinson’s treatment, Early diagnosis, and Parkinson’s genetics. See Supporting Table S1 for a full list of these engagement activities.

### 2.2. Ethical Considerations

This study received ethical approval from the Queen Square Ethics Committee (REC reference: 10/H0716/85, IRAS project ID: 20,769). Informed consent was sought for the analysis of users’ public interactions and engagement with the forum. We adhered to guidelines developed by the British Psychological Society on internet‐mediated research [27]. To protect the identity of forum users, usernames were omitted, as well as any personal information which risked revealing their identity.

### 2.3. Analysis

Thematic analysis was used to construct patterns within the textual data. An essentialist/realist theoretical framework was adopted which assumes a simple unidirectional relationship between the meanings and realities within participants’ data, since our aim was to understand how the forum was experienced by users [28]. Thematic analysis was inductive, constructing themes directly from the forum messages. The analysis was carried out in accordance with the approach outlined by Braun and Clarke [28, 29]; this was an iterative process, which involved repeatedly moving from the raw data and constructed themes, and vice versa.

Forum data were exported, anonymised and saved to password‐protected Excel files for analysis. Two authors (LJS and JJ) first familiarised themselves with the data by reading all forum posts, and making initial notes on their observations of the topics expressed and any potential patterns. Secondly, the authors (LJS and JJ) individually coded patterns within the dataset to describe basic interpretations. Descriptive/interpretative codes were manually assigned to the text, often assigning multiple codes to the same segment. After all data had been double‐coded, the researchers (LJS and JJ) met to discuss and compare ideas about which components were reoccurring or emphasised and discussed how pertinent codes could be collated to establish initial themes. These initial themes were then presented to the wider research team alongside example quotes for their feedback. Finally, themes were reviewed and revised to ensure they captured the data set appropriately. The SRQR Checklist [30] has been used to report this qualitative study (see Supporting Information S2).

### 2.4. Reflexivity

All authors are involved in PD research. LJS is a Health Psychologist; JJ and AG are Research Assistants; XL and BH are Postdoctoral Researchers; AS, CS, LP‐C and AN are academic Neurologists; and ADS is an academic General Practitioner. AN and AS developed and lead the PREDICT‐PD cohort study. Researchers leading on the qualitative analysis (LJS and JJ) were not previously involved in PREDICT‐PD or in moderating the forum, supporting a position of open enquiry. Both analysts engaged in reflexive practice by documenting their analytical decisions and meeting regularly to discuss their own subjectivity within the research process [31].

## 3. Results

### 3.1. Sample Characteristics

Based on postings, some users identified themselves as people with prodromal PD (including hyposmia, iRBD, patients with genetic variants), family members of people with PD and healthy adults with an interest in PD. Demographic data were not collected within this study but could be matched for most users with previously collected data from the PREDICT‐PD database (see Table 1).

**TABLE 1:** Matched demographic information available from the PREDICT‐PD database.

Sample characteristics of participants (*n* = 218)	Values
Age (years)	
Known age, *n* (%)	187 (86)
Mean (range)	67.59 (25.84–83.60)

Gender	
Known gender	193 (89)
Male, *n* (%)	80 (37)
Female, *n* (%)	113 (52)

Risk of Parkinson’s disease (PD)	
Known risk, *n* (%)	187 (86)
Risk score^∗^ (odds ratio, odd 1:*x*)	1:67

^∗^Based on PREDICT‐PD risk algorithm which incorporates demographic information, environmental exposures, and early symptoms [32].

### 3.2. Forum Characteristics

Threads included conversations initiated by users of the forum (20 threads, 133 posts written by 99 individual users) and the research team (14 threads, 231 posts written by 158 individual users). Further information on the network of postings and distribution of replies on the forum is reported elsewhere [26].

We initially looked separately at the content of posts within threads initiated by forum users relative to members of the research team. Those initiated by users tended to disclose more personal information, offer social support and connect over shared experiences. Discussions initiated by researchers offered a space for users to exchange opinions, advice and knowledge, and address political and societal issues unique to PD. We chose to combine postings to provide a more comprehensive overview of the data.

### 3.3. Thematic Overview

Four major themes were constructed across the dataset: Shared experiences, Proactive approach, Forum usability, and Recognition and awareness. Themes and their corresponding subthemes are presented in Table 2 and described below with illustrative excerpts. An ellipsis indicates where text has been removed for brevity. For clarity, each individual quoted below is given a unique identifying number (P1‐P50).

**Table 2:** Overview of themes and subthemes across the dataset.

**Shared Experiences**	**Proactive Approach**

*Establishing Connections*	*Symptom Management*
• Self‐disclosure	• Seeking experiential knowledge
• Reaching out	• Practical advice and suggestions
• Motivations for joining	• Medications–finding a solution
*Symptom Appraisal*	*Personal Responsibility*
• Describing symptoms and their impact	• Origins and motivations
• Seeking feedback	• Behavioural and lifestyle changes
• Symptom interpretation	*Information and Resources*
*Emotional Impacts*	• Desire to improve understanding
• Symptom‐related worry	• Explaining concepts
• Emotional burden	• Keeping informed
• Validation and normalisation	
*Route to Diagnosis*	
• Diagnosis experiences	
• Diagnostic delays	

**Forum Usability**	**Recognition and Awareness of PD**

• Technical issues	*Healthcare*
• Reaching others through threads	• Timely diagnosis and treatment
• Forum design	• Risk profiles in healthcare records
• Accessibility of the forum	• Individualised care
	*Research*
	• Patient and public involvement
	• Diversity in PD research
	• Future research topics
	*Political and Societal*
	• Societal awareness
	• Public campaigns
	• PD‐risk disclosure
	• Funding agenda

*Note:* Themes are presented in bold, with subthemes in italics.

### 3.4. Shared Experiences

#### 3.4.1. Establishing Connections

Most users introduced themselves by disclosing personal information including their age, whether they or a family member were experiencing symptoms, and how this had affected them. Self‐disclosure enabled users to reach out, develop connections and find others with shared experiences.

*I’m [age] and have absolutely no sign of Parkinson’s, but three close members of my family died of it… All a bit close to home, and I joined this hoping I’d be able to add something to the knowledge about this disease. Anyone similar out there? (P1)*

*My mother developed PD in her late 70s and lived to age 90 with mild PD symptoms. My brother currently aged 88 was diagnosed 3 years ago with rapid development of symptoms. (P2)*

Other users then validated their experiences (confirmation, approval) and provided feedback (sharing their own personal information and experiences). This created a dialogue and fostered a sense of community.

*I am similar. My brother had Parkinson’s, and I watched his decline… if I can help in any way, I feel I do so in memory of my brother who died aged 68.* (P3)

*I too have had a diagnosis of REM* Sleep *disorder some 4 years ago.* (P4)

Some users also introduced themselves by stating their motivations for joining the forum and what they hoped to gain. This commonly included a desire to advance research, learn more about PD, connect with similar others and because they know someone affected by PD.

*My background and motivation are very similar. My brother lived with Parkinson′s for over 30 years before he died and was a* committed *supporter of research into the condition, so I′ve wanted to help where I can.* (P5)

#### 3.4.2. Symptom Appraisal

Members used the forum to discuss symptoms and their impacts with others who understood their experiences. Several users were empowered to set up symptom‐related threads to catalyse engagement related to a topic of personal interest. Conversations commonly unfolded by users first providing detailed descriptions of their symptoms and describing how these impact daily life.

*Over the past year, I have noticed a deterioration in memory and smell senses. Smell - as a former Wine Judge, I stopped judging 4 years ago when symptoms got worse.* (P6)

*Names of things, people and places come and go… I find it increasingly difficult to hold a conversation too as a result.* (P7)

Many users tried to foster a dialogue by inviting responses from others. This often involved asking other users whether they shared their symptom experiences. The forum was used to seek confirmation and social validation from others. Users also invited feedback on how these symptoms were experienced by others (what this looks like for them).

*In the last 18 months, I’ve started* moving *and vocalising when dreaming, maybe once every 6 weeks or so… Anyone else experienced similar or can offer any reassurance?* (P8)

*Hello, I am almost 70 and for the* last *6 months have been experiencing certain symptoms. I was interested to see that you have a tremor and slow movements. Can I ask, do you find you are walking more slowly?* (P9)

Conversations then moved onto the underlying nature of symptoms experienced. Users wanted to understand whether their symptoms were an early indicator of PD or related to other causes. They interpreted the different sensations experienced and evaluated potential contributing factors (e.g., ageing, hearing loss, concomitant conditions).

*Memory loss- Has been much quicker recently, forgetting names of plants, what I′m trying to say, what I’ve gone to get… I attribute some to my deafness, despite 2 hearing aids.* (P6)

Some sought others’ opinions to help them understand how their symptoms might progress. While users responded to these requests with suggestions, they recognised the need for professional input.

*Even though I have no expertise in this field, I think your GP needs to see your husband for himself or herself so they can make their assessment based on their own observations.* (P10)

#### 3.4.3. Emotional Impacts

Feelings of symptom‐related worry were commonplace, including concerns about the potential progression of symptoms and whether these were a manifestation of PD.

*Memory loss- Has been much quicker recently, forgetting names of plants, what I’m trying to say, what I’ve gone to get… I attribute some to my deafness, despite 2 hearing aids.* (P6)

Those with a family history of PD were also concerned about family members developing the condition.

*My sons are already anxious* about *having PD because they see similarities in my symptoms and in my late father, their grandfather. They are changing their lifestyle already.* (P12)

*My husband developed Parkinson’s and survived for 6 years after the first diagnosis. His sister, 9 years his junior, also developed it 2 years after him but died within 3 years… I am concerned if it is hereditary.* (P13)

In response, the forum served as a space to provide and receive emotional support. This included normalising emotions experienced as an understandable reaction, reassuring others that they were not alone, and expressing empathy through words of comfort.

*My husband did this too… These vivid, bad dreams went on for some time and then subsided. Do please discuss it with your GP. Your poor wife must be equally anxious.* (P14)

*While RBD increases the* risk *of d*e*veloping PD in the future, not everyone who has RBD goes on to develop PD, so no point worrying. Good luck.* (P15)

In return, users acknowledged each other’s posts, expressing gratitude for their input. This fostered a compassionate community who expressed concern for each other’s well‐being.

*Occasionally my husband* tells *me that I have been shouting in my sleep… After reading the other posts I am going to monitor the circumstances in which this happens. Had no idea there were any possible connections with anything diagnosable so thank you.* (P16)

#### 3.4.4. Route to Diagnosis

Diagnosis experiences formed an important part of discussions. Users described suggestions from doctors about what their condition might be and for how long their symptoms had presented. It was common for other members to reciprocate and share their own experiences as a response to a story shared or question asked by a user.

*I have similar issues, in the past I have head butted and kicked my wife while dreaming that I am playing football… I have been sent to the sleep unit and been diagnosed with sleep apnoea.* (P17)

*I too had a tremor and went to see a world leading neurologist… He diagnosed Essential Tremor and prescribed Propranolol, which I have taken ever since.* (P18)

They shared experiential information on their care journey, including which professionals they spoke to, and medical investigations and tests undertaken. Moreover, some users provided insights on what to expect from medical investigations and what support to request.

*I’ve had exactly the same experiences and symptoms, which following a sleep study was diagnosed as RBD… Talk to your doctor as I think you need a specialist referral for a sleep study to confirm if RBD. Nothing to it, just a night’s kip in hospital/ clinic while wired up with electrodes everywhere and videoed while asleep.* (P15)

Diagnostic delays were common, including long waiting lists and difficulties accessing a specialist. Personal factors also contributed to delays in seeking help and getting a diagnosis. Anxiety about receiving a diagnosis, concerns around being misdiagnosed and uncertainties of the implications of being diagnosed (e.g., on driving) were discussed as reasons for delaying contact with health services.

*Waiting lists for any assessment are so long in our area - getting ‘into the system’ asap would be preferable so that appropriate monitoring/advice/medication can be given in a timely manner.* (P19)

*As I now know this can be an early predictor of PD, I’m a little anxious, but nervous about going to the doctor to discuss.* (P8)

### 3.5. Proactive Approach

#### 3.5.1. Symptom Management

Users actively sought experiential knowledge from others. They initiated threads to request advice on how to better manage their symptoms. Members appeared to value this support because it came from people with shared health experiences.

*I have found recently that despite the medication the incidents are happening more frequently … I wondered if anyone else had experience of RBD and if they have found a way of reducing or removing their symptoms. I would love to hear.* (P20)

*I am experiencing frequent bouts of freezing which feels like my feet are nailed to the floor and I cannot move… answers from this forum to how to remedy this lifestyle limiter will be welcomed.* (P21)

Other users responded by sharing the strategies they had implemented, including home adaptations, exercise, peer support and keeping mentally active. Users described how adopting these strategies had helped to reduce their symptoms, restore a sense of self‐control and aid daily coping.

*I have a ‘crash pad’ foam mattress on the floor alongside the bed and padding on the furniture near the bed to minimise risk of self-injury from any violent actions.* (P15)

*I now manage it also with yoga x 4 per week and plenty of serious exercise… Latest webinars and research have stressed the importance of exercise which is great news to me.* (P22)

Others referred to medications, sharing what had/had not worked for them (or someone close to them). Users showed willingness to try different approaches and were motivated to find an effective solution.

*Does anyone have experience of using CBD to treat RBD? I take a combination of Clonazapam and Melatonin at night which* keeps *it almost under control. But I would love to replace the Clonazapam with a more natural substance.* (P20)

Some also provided information on services to help people cope and live well.

*I would just like to say that,* having *a distant acquaintance with Parkinsons, I think that support is more available than it may have been in the past, especially from Parkinson’s UK. Best wishes to you.* (P23)

#### 3.5.2. Personal Responsibility

Users expressed a notion of personal responsibility for their health, taking ownership to manage potential risks of PD and stay well. For some, the origin of this responsibility came from the self and encompassed striving to become the best possible version of themselves, attaining important life goals and planning for the future.

*I would like to know about PD even if there is not a cure… I would be able to take some personal responsibility to keep myself as well as possible for as long as possible and make life plans.* (P24)

*Parkinson’s is not a terminal diagnosis. It is not the proximal cause of death but the distal. That’s why it is important to try and stay fit and as active as possible.* (P25)

For others, this responsibility was influenced by personal relationships, including family members. By taking responsibility for their health, they hoped to reduce the impact and burden on loved ones.

*Having some control over the quality of one’s life is critical and important. Having seen a few people develop MS and the effects (good and bad) it had on their families, having those conversations earlier does enable hard decisions about the future to follow.* (P26)

Discussions also covered what it means to take personal responsibility. Users shared the proactive lifestyle changes they had made. These focused around physical exercise and diet.

*I take regular vigorous exercise,* have *a very healthy diet, drink in moderation at weekends only, weigh a bit less now than when I got married. So, I′m doing all the right things.* (P7)

#### 3.5.3. Information and Resources

Users frequently asked questions and actively sought out information to enhance their understanding and knowledge on a diverse range of topics relating to PD.

*Occasionally my husband* tells *I have heard that Parkinson’s affects more men than women. Is this true? Is there data to show this?* (P27)

*Occasionally my husband* tells *I was interested to read about the new skin swab test, anyone with any knowledge on this?* (P28)

Questions were also answered by forum users who explained concepts based on their own knowledge and research. Users showed awareness of the complexity of PD and used careful phrasing to indicate that their knowledge was drawn from personal experience rather than professional expertise.

*My understanding is that there are multiple genetic mutations that are involved but they have to be triggered, and the trigger is unknown. It is possible to have faulty genes and never develop symptoms… Hope this helps.* (P25)

External sources of information including websites and research papers were shared to provide additional detail. This knowledge exchange helped users to keep informed and equipped them with knowledge to aid their decision‐making and self‐management. Users valued information from a credible source or with a scientific evidence base.

*This website has some suggestions that might be of use to you. I hope you find something helpful.* https://www.apdaparkinson.org (P29)

*Only solid research with a viable base* can *provide answers with any degree of certainty.* (P30)

### 3.6. Forum Usability

On one occasion, there was a technical issue with the forum which caused users problems when logging in. The forum was used as a space to log these issues and update one another once these had been resolved.

*I am having the same problem finding a username - everyone I try says ‘name taken’. Surely not this soon after publishing the forum!* (P31)

Moreover, some users were concerned about whether others could see their replies. It was important to users that dialogues could continue.

*If someone replies to a post in one of the threads, can the original poster see that the reply is meant for them? I’ve replied to a couple* of *posts and the information says that I’ve replied to myself.* (P10)

When users reported difficulties interacting with the forum, others were quick to share tips and advice.

*I’d just like to add that in order to reveal the Logout option on the LH side of page, that one has to be viewing their tablet etc. in landscape view!* (P32)

Some users expressed concerns about the accessibility of the forum. They explained how PD symptomology (including cognitive problems and tremor), technology access and proficiency could prohibit engagement with the forum. They were concerned that some might be excluded or dependent on others to access.

*Online social contact is impossible for people with Parkinson’s to access independently. My husband is unable to use a computer now, so I have to be present to put him online and deal with any technical issues. So, this doesn′t enable him to have normal contact with others and is, for me at least, very frustrating as I need to be* around *24/7.* (P33)

*I sometimes think that there is also a cost feature, as not everyone can afford the availability of internet connections.* (P34)

### 3.7. Recognition and Awareness of PD

#### 3.7.1. Healthcare

The forum was often used as a space to discuss difficulties in accessing care for prodromal PD. This was frustrating for those experiencing symptoms, as since these were not recognised as an early indicator of PD, no treatment was available. Users thought research findings had not yet been translated into healthcare settings and advocated for a pathway that recognises prodromal symptoms and provides timely preventive treatment.

*I have heard that Parkinson’s affects more men than women. Is this true? Is there data to show this?* (P27)

*I was interested to read about the new skin swab test, anyone with any knowledge on this?* (P28)

Users thought it would be useful to have risk‐related information including prodromal symptoms, genetic risks and family history flagged on patients’ medical records to facilitate early detection amongst healthcare providers. Flagging this information was also thought to aid shared decision‐making.

*I think there is a point in knowing risk exists for people experiencing symptoms they or their doctors might* ignore*, or if there′s something that can be done to reduce risk, or by co-operating with others to reduce risk.* (P36)

I thin*k it would be useful to have it on record; in case there are things that I/medical professionals should be looking out for.* (P37)

Users recognised PD as a complex condition that affects people differently. Individual differences were also discussed in relation to PD risk. Users understood that not all those at‐risk would go on to develop PD. They appreciated that people would differ on their perspectives of PD risk including disclosure of this information and how they act on this.

*Ask anyone about their Parkinson’s and you’ll get just as many different answers. Each person’s symptoms are different.* (P38)

*Knowing that there is a chance of a diagnosis of Parkinson′s potentially on the horizon I imagine some people might be extra careful to avoid potential triggers and others would throw caution to the wind and indulge in any risky behaviours before they became incapacitated. No two people are alike. I fall into the risk-averse category.* (P29)

Users therefore advocated for an individualised approach to healthcare that recognises patients’ unique circumstances. This involves giving patients opportunities to discuss their health needs, beliefs and preferences and engaging them in their care.

*Other than that, generally* favourable *to having risks explained, though probably needs variable information depending on severity of RBD, and age and family situation of subject.* (P39)

#### 3.7.2. Research

The forum was also used to discuss issues and aspirations around PD research. Users wanted to receive feedback on studies they participated in and advocated for PPI within research.

*I’ve found contributing to citizen science really worthwhile… especially where we get feedback on the results of studies and how they are published.* (P40)

Users recognised that people are affected differently by PD and that some groups have a higher incidence of disease. Some therefore stressed ‘*diversity in research is especially important in Parkinson’s’* (P38), both in terms of the people taking part and the research team conducting the study. By recognising and promoting diversity, users hoped research could help to uncover the reasons behind health disparities and provide meaningful insights.

*Diversity of volunteers participating in research such as this is essential… without diversity you can surely only assist one section of the community with certainty.* (P41)

*Diversity and sex should also be noted. I think there should also be diversity in researchers.* (P42)

Users discussed which topics they thought should be researched. They recognised the need to balance research across both prevention and treatment to reduce the number of people diagnosed and address the needs of those living with the condition. A broad range of topics were highlighted including lifestyle, environmental, genetic and social factors.

*In my opinion we should prioritise research into prevention in the hope that for the future the scale of the problem will be decreased.* (P43)

*There is a balance required between treatment and prevention. However, designing and devising treatment will often lead to prevention therefore perhaps there is an edge towards investing in treatment.* (P44)

Users were engaged with research and held optimism for the future. Some also proposed ideas for potential projects which they felt could offer novel insights.

*I wonder if studying people who do not show Parkinsons′ symptoms might be just as important as studying those who show the symptoms… perhaps AI and big data might be the only way we could figure out what questions to ask.* (P45)

#### 3.7.3. Political and Societal

Users advocated for more societal awareness of PD. There was a perception that PD had received less attention relative to other health conditions. Better recognition of the range of PD symptoms was also highlighted, particularly symptoms that are less visible (e.g., cognition, sleep, hyposmia), as well as how to support people to live well for longer. Public campaigns were suggested to achieve this via adverts, articles and educational sessions in schools.

*Articles in national newspapers would make people more aware of the disease, and free ads for the charities that support it. We see a lot of adverts for cancer charities so it would be beneficial for people to be made more aware of other areas of need.* (P46)

*There are many diseases including Parkinson’s and Dementia and others of which many people would benefit from school education input.* (P47)

Users also discussed their concerns around information on PD‐risk disclosure being shared. Some were apprehensive about this being used to discriminate against people to increase their insurance policies or restrict their driving.

*If people knew they were likely to get PD they would have to declare this on any health insurance and travel insurance policy, and this would put up the premiums. I think we can have too much information.* (P42)

*If I were at risk of any illness, I would prefer to know so that I can take the necessary action if further symptoms develop. Living in a rural area, my biggest concern would be an over-cautious doctor who prevented me from driving when this wasn′t necessary.* (P48)

Users therefore thought education and guidance should be provided to these sectors to ensure informed and fair decisions.

*In my view knowledge is power and therefore I would always want to know in full about every aspect of any diagnosed condition. However, the ′power′ extends to the likes of insurance companies who could potentially be keen to refuse insurance based on the diagnosis on a just-in-case basis for future problems… Education of others, such as insurers, is required.* (P29)

The forum was also used to contextualise PD within the broader societal and political climate, with users discussing how public funding is allocated. Comparisons were made to COVID‐19 which was perceived as being heavily funded. Users thought the prevalence, rising incidence and impact of PD warranted further prioritisation.

*Yes, it’s always about decision-making and those who set the agenda hold all the keys… Any additional funding would be welcome in this and other closely related areas.* (P49)

*Considering such a lot of successful research and vaccination work has been prioritised for Covid … it shows how it is possible to achieve amazing results in a short space of time with a lot of money thrown at it by government and pharmaceutical companies. Considering the numbers of people diagnosed every year with Parkinson’s surely this sort of priority could be put on finding solutions to treat this dreadful disease.* (P50)

## 4. Discussion

Our study offers insights into how a digital social forum embedded within the PREDICT‐PD portal was used. Users appeared to value and engage with the forum because it provided opportunities to connect with others who share their health experiences and could be considered peers [34]. They sought validation, shared experiences, exchanged information and discussed political and societal issues related to PD. These results align with the core features of online peer support outlined in the literature, namely, emotional and informational support [35]. Users exchanged emotional support through empathy, compassion and reassurance. Emotional peer support can provide comfort, enhance self‐esteem and reduce loneliness and isolation [16, 35]. More generally, the forum offered a space for users to disclose personal information and tell their story which can have a therapeutic effect in itself. Practical advice, resources and information were also exchanged on the forum to help users expand their knowledge base. This informational support can help give users a sense of control over their situation and reduce uncertainty to enable better decision‐making [35, 36].

Users aimed to achieve personal well‐being and shared experiential knowledge to develop coping strategies to manage existing symptoms, or make lifestyle changes to alter the course of PD. For example, several users sought to learn about iRBD and expressed an interest in medical management and complementary therapeutic strategies. Lifestyle changes included maintaining sleep hygiene, keeping physically active and making healthy diet choices. Research supports the use of these in PD and prodromal PD (7, 38), confirming that online peer communities may provide reliable solutions to promote health and well‐being. Users expressed a notion of personal responsibility for their health, taking ownership and adopting a proactive approach to manage potential risks of PD and stay well. Our findings can be understood using the framework proposed by Björk et al. [38] which outlines where the responsibility for health comes from (within yourself, relationship to specific others, relationships with generalised others) and what this means. Here, responsibility for health appeared to be motivated by a drive to stay well for the individual’s own benefit, but also to reduce burdens on loved ones and wider society. This was expressed by implementing active measures to sustain and improve health (including lifestyle changes and symptom management), and seeking and accepting help in health from peers, family and healthcare professionals [38].

This study also offered unique insights into the complex ethical issues surrounding the identification and management of prodromal PD. Users with symptoms or a family history of PD were often worried about developing the disease. Moreover, some users were anxious about speaking to a healthcare professional for fear that they would receive a positive diagnosis, while others were eager to obtain a diagnosis to plan for the future. Perspectives on risk disclosure varied, most were willing to know and felt that being aware of their risk would allow informed decision‐making. However, others only wanted to have their risk disclosed if there was something that could be done to mitigate this. These findings align with previous surveys conducted with iRBD patient groups [25, 39] and support an individualised approach to PD‐risk disclosure that takes into account patient’s preferences, opinions, knowledge and emotional well‐being [25]. Users also expressed concerns about who their risk might be shared with and what impact this might have on their driving licence or insurance policy. Eaden et al. [40] highlight the need for realistic information about screening and risks to be offered to patients so that they understand what can and cannot be achieved by current science. Our findings suggest this information should extend to broader society to protect against discrimination within employment, healthcare, driving and insurance contexts.

Misinformation and online misbehaviours (e.g., trolling, harassment, abuse) present barriers to online peer support and can lead to negative health outcomes. Previous studies with larger, open communities have documented the presence of these behaviours in PD (42, 43), multiple sclerosis [43] and dementia [44]. These were absent in this study. This is likely due to the study’s design, which was based on an established, closed cohort of individuals motivated to support research in this field. Posts were carefully phrased by users to convey that information being shared was based on their own experience, knowledge or opinion. Users also acknowledged the limits of their knowledge and advised others to seek medical input. Previous research suggests having a homogeneous group is important to the success of an online peer support group [34]. The fact that users of the forum were a closed group of participants from the PREDICT‐PD research study may therefore have helped to foster a supportive online community.

Venkatesan et al. [41] also show that misinformation depends on the clarity of the questions posted by the user who created the thread. Here, posts from the research team were phrased as questions designed to facilitate discussion. Posts initiated by researchers may therefore have served as examples for user‐initiated threads and helped them to compose their questions [45]. Moreover, these researcher posts allowed users to exchange knowledge, ask questions and share their ambitions for the future. The involvement of professionals may therefore facilitate factual interactions, but user‐initiated threads remain important for sharing personal experiences and exchanging social support [45]. Our study suggests combining both these perspectives can help sustain a vibrant online community.

Digital literacy has also been shown to present barriers to online peer support in PD [47]. In the current study, some users demonstrated digital proficiency, advising others on how to use the forum (e.g., change font style or sign out of the platform) and providing suggestions on how to make the forum compatible for use on different devices. Moreover, when a technical issue occurred during the pilot, users posted to report errors and update others when problems had been resolved. However, users were also aware that others may be excluded from engaging with the forum because of debilitating PD symptoms and limited digital literacy. Progressive iterations of the forum should now be developed using a co‐design approach to ensure the forum technology meets the needs and expectations of users with varying degrees of PD symptomology and digital literacy [47].

### 4.1. Strengths and Limitations

This study is to our knowledge the first to explore online peer support in an at‐risk PD cohort. Harnessing the PREDICT‐PD platform allowed us to create a vibrant digital community including people with prodromal PD, family members of people with PD and healthy adults with an interest in PD. Moreover, embedding the forum within PREDICT‐PD fostered a safe space for social engagement via a closed community, moderated by the research team.

This study has several limitations. Firstly, since all users were already volunteering in PREDICT‐PD, their perspectives and experiences may differ from the wider community. Moreover, moderation and postings by the research team may have affected interactions between users and restricted what they felt comfortable to share [48]. Secondly, since only public posts were analysed, we were unable to explore the experiences of users who read the posts but did not interact, also called ‘lurkers’. Future research could include interviews with nonactive users to explore their reactions to the forum, reasons for lurking behaviour, interpretations of forum content and any benefits derived from using it [49, 50]. Finally, we were able to match participants’ social engagement on the forum with previously collected PREDICT‐PD data for some but not all participants. This meant that posts were analysed collectively providing a broad overview of the dataset. Future research should explore how experiences and perceptions differ between stakeholder groups (e.g., individuals with prodromal or diagnosed PD, family members or interested members of the public).

## 5. Conclusion

The online forum fostered a vibrant peer support community where users could connect with others to share experiences and exchange emotional and informational support. Currently, there is little support available for people with prodromal PD, and this forum provided access to a wide range of people who might otherwise be difficult to find in their local areas [35]. Low social engagement is a risk factor for neurodegeneration, while high social engagement can be neuroprotective. Future studies should therefore establish whether participating in an online forum can increase social engagement in prodromal PD. The relatively low costs of providing and maintaining an online forum and the voluntary basis of its participants’ contribution are likely to make such an intervention cost‐effective [17]. Further research is needed to develop accessible technologies that meet users’ needs and to examine the longitudinal impact of online peer support on PD risk.

## Funding

This study was funded by Barts Charity, MGU04579; and PREDICT‐PD was funded by Parkinson’s UK.

## Conflicts of Interest

The authors declare no conflicts of interest.

## Supporting Information

<< Table S1. Initial posts from threads initiated by the research team designed to simulate discussions.>>

Description: Content of weekly engagement activities posted by the research team (including PD‐related research, facts and queries) to stimulate discussions.

## Supporting information


**Supporting Information** Additional supporting information can be found online in the Supporting Information section.

## Data Availability

The data that support the findings of this study are available upon reasonable request from the corresponding author. The data are not publicly available due to privacy or ethical restrictions.
