# Supplementary material for: The Use of a Digital Social Forum in a Parkinson’s Disease Risk Cohort: A Thematic Analysis of Forum Messages
Source: Parkinsons Dis. 2026 Feb 13;2026:7537110. doi: 10.1155/padi/7537110 (PMC12903991; doi:10.1155/padi/7537110)
Supplement: Supplementary file 1 — Supporting Information Additional supporting information can be found online in the Supporting Information section. [file PADI-2026-7537110-s001.zip › Supplementary Materials Table S1.docx]

**Supplementary Materials**

Table S1. Initial posts from threads initiated by the research team designed to simulate discussions.

| **Topic** | **Post** |
| --- | --- |
| Team introduction | Welcome to the PREDICT-PD forum. We will be testing this out over 12 weeks. We hope you find it a safe place to discuss topics from the things we post, share experiences and interact with other participants.  When using the forum please adhere to the following guidelines:   - Be respectful to other members at all times. - Do not post inappropriate or disrespectful comments, images or links. - Do not post advertisements or spam. - Do not post personal information e.g. full names, personal contact information, addresses or passwords.   The forum will be moderated to ensure all guidelines are adhered to.  If you have any questions, please email: aneet.gill@qmul.ac.uk  We look forward to seeing you each week with a new post!  The PREDICT-PD Team |
| The rising burden of Parkinson’s | We often hear that the world is getting older and people are living for longer. It is perhaps unsurprising that certain diseases that are associated with aging are getting more common. This is true for some of the neurological diseases like Parkinson’s and dementia.  Compared to dementia the number of people with Parkinson’s is much smaller. There are an estimated 50 million people worldwide with dementia, whereas only about 7 million have Parkinson’s. As brain diseases associated with age, you might expect Parkinson’s and dementia to be increasing at similar rates. But this does not seem to be true.  Data from the Global Burden of Disease study and similar research suggest that dementia increases are almost entirely due to aging. Once we take account of age, actually there is some suggestion that dementia might be falling. This is probably because some of the risk factors for dementia overlap with the risk factors for heart disease, stroke and cancer. As we get better at preventing these diseases, we have also had an effect on dementia.  In contrast, Parkinson’s is increasing even after we take account of age. This means that something or several things are contributing to Parkinson’s other than age.  Part of our work is on identifying the things that cause Parkinson’s and might explain an increasing burden over time.  Question: Why do you think that Parkinson’s might be on the increase? What should be done to stem the tide of Parkinson’s? |
| Parkinson’s treatment | There is no cure for Parkinson’s. Although we know that losing brain cells in certain areas is responsible for Parkinson’s symptoms, we don’t yet know how to stop those cells from dying. Despite this, we do have very good drugs that treat the symptoms of Parkinson’s.  These drugs work by doing the following things: increasing the amount of dopamine in the brain, acting as a substitute for dopamine by stimulating the places where dopamine acts and blocking the breakdown of dopamine. Although none of these stop the underlying disease, treating the symptoms can dramatically improve quality of life for patients.  There is no correct answer for when we should start treatment in people with Parkinson’s. The decision is made for each person depending on the severity of their symptoms and how much they interfere with daily activities.  As time goes on, more tablets tend to be added to the treatment plan. Sometimes alternatives to tablets must be considered. These include injections of drugs under the skin or directly into the gut, or sometimes brain surgery.  Aside from drugs, there are many other therapies that can help. People with Parkinson’s often require management by physiotherapists, specialist nurses, speech and language therapists, occupational therapists, psychologists and dieticians.  In addition, living a ‘brain healthy’ lifestyle is very important and involves keeping the brain and body active, and having a well-balanced diet.  Question:  Should we be prioritising prevention of Parkinson’s or better treatment for patients that already have Parkinson’s? |
| Risk disclosure and ethics | REM sleep behaviour disorder (RBD) is a condition in which people have vivid or ‘action-packed’ dreams. People tend to ‘act-out’, ‘fight’ or ‘shout’ during their sleep.  RBD is pretty rare and to diagnose it properly a sleep study in hospital is required. It is associated with an increased risk of later developing Parkinson’s or related condition. Whether and when this occurs is very variable. RBD may occur years or even decades before other neurological diseases.  There are currently no treatments that prevent or reduce the progression of RBD to other conditions. Therefore, at the point of diagnosis, there is controversy about what information should be disclosed about the potential future implications.  Questions:  Imagine you have been diagnosed with RBD. What preferences would you have about the information you receive regarding links with Parkinson’s and related conditions. How and when should these discussions take place (if at all)?  What other factors might impact your decision about what, how and when to receive this information?  Would your decision change if there was a way to stop RBD progressing to Parkinson’s? |
| Biomarkers | Parkinson’s is diagnosed in the clinic once motor symptoms have begun to show. At this point up to 50% of the brain cells that are damaged in Parkinson’s may have already been lost. Reliable markers are needed to make the diagnosis at the earliest possible stage, which in turn will give new drugs the best chance of working well.  The term ‘biomarker’ reflects any marker of the biological changes in Parkinson’s. Biomarkers can be found in scans of the brain and in body fluids. Lots of research is ongoing into markers of Parkinson’s in spinal fluid and blood. Samples from spinal fluid are beneficial because they are more likely to reflect directly what is happening in the brain. However they require a lumbar puncture, which is invasive and off-putting. As an alternative, other research is focussed on the measurement of biomarkers in blood.  The field of Parkinson’s has advanced rapidly in the last 5-10 years, and we are ever closer to having reliable biomarkers. Lessons are being learnt from the field of Alzheimer’s, where through a vigorous standardised biomarker framework and extensive research, some biomarkers are starting to be used in clinics to aid diagnosis.  Questions:  How important do you think it is that research focuses on biomarkers of Parkinson’s?  What is your opinion on whether research should focus on biomarkers in spinal fluid (requiring a lumbar puncture) or blood (requiring a normal blood test) or both? |
| Early vs. timely diagnosis | One of the aims of PREDICT-PD is to identify people at the earliest stages of Parkinson’s before disability occurs. There is no cure for Parkinson’s and we think part of the reason is that people get diagnosed too late. If we could identify people earlier then the drugs that we hope will slow down Parkinson’s would have the best chance of working. However, while earlier diagnosis is one goal, it is not the only consideration.  A timely diagnosis is a different notion and what is timely will mean different things to different people. For example, if there is a drug that slows down Parkinson’s, an earlier diagnosis will probably mean the same as a timely diagnosis. On the other hand, if there is no drug to slow down Parkinson’s then timely could mean knowing in advance so that you can prioritise all of the things you want to do OR alternatively only finding out when you have symptoms so that you can get treatment for the symptoms.  Timeliness requires us to take account of both availability of treatments and individual preferences.  Questions:  What does timely mean to you? Are you the kind of person who would want to know that you were at risk of a disease even if there was no treatment? Or are you the kind of person who would only want to know if something could be done about it? |
| Increased diversity in research | Health research is often done with a small proportion of the overall population, with the goal of providing an effective treatment or intervention for the population as a whole. However, without a diverse group of individuals participating in research, scientists will not know if their results can be applied to all people equally. Diversity in research means that people of different ages, different racial and ethnic groups, and both men and women participate in research studies.  Diversity in Parkinson’s disease (PD) research is equally important. Participants in PD research should reflect the diversity of culture and conditions, considering race, ethnicity, gender, age, etc.  Certain minority groups have a higher incidence of diseases, such as diabetes, obesity, asthma, and CVD, than Caucasians. By inclusion of these minority groups, researchers will be able to understand biological, social, environmental and other factors which contribute to these disparities in health. This will also allow us to identify how these diseases can affect PD. Without participation of diverse groups, we would not know the existence of and the reason behind these disparities.  Designing trials with more inclusive procedures that encourage minority groups to participate can enhance medical understanding of race and ethnic based variation in response and reduce outcome disparities. This will result in researchers making medical products and treatments that will be ultimately used by the general population.  Considering logistical and financial resources during trial design, and effective communication with the participants and their families to demonstrate the value of clinical trials and the importance of research can make trails more accessible to underrepresented groups.  Partnership with care providers, religions leaders and community based medical centres may also be useful to reduce inequalities and get more diversified results from research trials. These centres have a keen understanding of their local community. They care about their patients and often can be research trials' biggest recruiter and cheerleader.  Question:  How important do you think diversity is in gaining a holistic overview of Parkinson's disease? |
| Smell Loss in COVID-19 and risk of neurodegeneration by | A significant amount of research over the last 18 months has been understandably directed at COVID-19. Because the virus is so new, very little is known about the long-term implications of having been infected with COVID-19.  Interestingly, one of the key symptoms of COVID-19 has been loss of or change in sense of smell. Though it may sound peculiar, smell change is also an early symptom of some common neurodegenerative diseases including Parkinson’s and Alzheimer’s disease.  A recent study found that a third of people had been diagnosed with either a neurological or a psychiatric condition within 6 months of having COVID-19. There could be many reasons for this. The experience of being very unwell can have consequences on mental health and the virus can affect many body systems, including the brain. One of the study’s conclusions was that the relationship between COVID-19 and future Parkinsonism should be studied over time. Could COVID-19 be a risk factor for developing Parkinson’s disease in the future?  Loss of sense of smell is a complex process that is not yet fully understood. Increased focus on this symptom may help us understand the role that smell loss plays in the development of neurodegenerative diseases, as well as COVID-19.  Question:  How do you think research into COVID-19 could help us understand the causes of Parkinson’s Disease? |
| Parkinson’s genetics | The role of genetics in Parkinson’s disease has a complicated history. Until the 1990s, the scientific community was unsure that genetics had any role at all. In 1997 a study found that individuals in families with a lot of Parkinson’s had mutations (changes in the genetic sequence) of the *SNCA* gene. Since then, multiple genes and genetic locations have been linked to Parkinson’s.  There are genes that can cause Parkinson’s with a single mutation. These are called the ‘monogenic’ forms. Mutations in *SNCA* is one of the monogenic causes of Parkinson’s. *SNCA* encodes the alpha-synuclein protein, the protein that forms Lewy bodies in the brain. Lewy bodies are the hallmark of Parkinson’s. Other monogenic causes of Parkinson's include mutations *PRKN*, *GBA*, and *LRRK2*. Most of these are rare.  The majority of people with Parkinson’s do not have a monogenic explanation. Some changes in genes do not seem to cause Parkinson’s on their own, but rather seem to put people at higher risk than others. If someone has enough of these changes, in enough of their genes, it may put that person at higher risk of developing Parkinson’s. These mutations or changes are called polygenic risk factors. As of 2019, scientists have found about 90 different polygenic risk factors that increase the risk of Parkinson’s. We can estimate someone’s risk for Parkinson’s by calculating a ‘polygenic risk score’. The higher the score, the higher the risk that someone has for Parkinson’s. People in the top 25% of scores may have up to 6 times more risk for PD than those in the lower 25%.  Questions:   1. Do you feel that genetic risk for diseases is well explained by the medical and scientific community? If not, why not? 2. Do you think it will be useful for people to know their polygenic risk score for Parkinson’s? |
| Socialising online - impact on health | People who have stronger social networks appear to live longer. Recent studies suggested that lifestyle interventions such as reducing stress and increasing social interaction may be protective factors for diseases such Parkinson’s.  However, can we say the same about online social networks? The question is even more relevant now, with COVID-19 lockdowns that made face-to-face meetings difficult.  A study of 12 million people in California 3 years ago found that people who use online social media such as Facebook have lower mortality rates than those who do not.  A research team at QMUL recently conducted a public engagement activity exploring offline and online social interaction of people suffering with chronic lung conditions. Online social contacts provided valuable support when family and friends were not available (e.g. during hospital admissions). Participants displayed different traits, and some were more open to share information about their health than others. You can see a 1-minute video about this kind of public engagement activity here<https://bit.ly/3h7Yg6v>.  Social media platforms provide an opportunity to maintain social relationships, but also provide people with greater capacity to receive social support and encourage socially-motivated behaviours that may prevent illness.  Questions:    - What is your experience of online interactions on social media platforms? - Do you ever discuss health (an illness, if applicable) with your peers? |
| Linkage to NHS health records | There are many positive things to say about the National Health Service, but one special thing is the huge volume of health data that is captured about patients from primary and secondary care. Although health systems around the world gather data on their patients, there are very few that have the coverage the NHS gives us in the UK. This data has the potential to transform our understanding of disease (and health) and enhance research to develop better treatments and cures for Parkinson’s. However, there are important considerations given the highly sensitive nature of health data. These days data usage, processing and storage is governed by a set of regulations known as GDPR.  Many research studies obtain consent from their participants to link to their NHS health records data. This is usually done via the NHS number and in doing so it means that no identifiable information (e.g. name, date of birth, address) is exchanged.  The UK Biobank study set a precedent for doing this – participants were recruited at the beginning and answered questions, underwent scans, and donated samples. The information gathered at the beginning is linked to information about new diagnoses and outcomes in NHS health records. In PREDICT-PD we obtain consent from our participants to do the same thing. It will mean that we can better find out who gets Parkinson’s in the future, which in turn will make our prediction models more refined. All of which brings us closer to one day being able to prevent or delay the onset of Parkinson’s.  Question:  How do you feel about having your NHS health records data linked with your PREDICT-PD participant data? |
